# Silver-coated *Zea mays L.* nanocatalyst for efficient Azo dye photodegradation and antimicrobial applications

**DOI:** 10.1038/s41598-025-22961-9

**Published:** 2025-10-30

**Authors:** Walaa M. Abd El‐Gawad, Karim Elmaghraby, Ahmed M. El-Khawaga

**Affiliations:** 1 Polymers and Pigments Department, National Research Centre, Dokki, 12622 Cairo Egypt; 2 Department of Botany and Microbiology, Faculty of Science, Tanta University, Tanta, 31527 Egypt; 3 Department of Biotechnology, Chemistry and Pharmacy, University of Siena, Siena, 53100 Italy; 4 Department of Basic Medical Sciences, Faculty of Medicine, Galala University, Galala City, 43511 Suez Egypt

**Keywords:** Photocatalysis, Core-shell nanocatalyst, Zea mays L., Azo dye, Antibacterial activity, Environmental sciences, Nanoscience and technology

## Abstract

The increasing environmental issues and anticipated energy crisis highlight the urgent need for a cost-effective and efficient photocatalyst that responds to UV light for contaminant degradation. This work presents a novel approach to synthesizing *Zea mays L.*-loaded silver oxide nanoparticles (Ag_2_O) by chemically depositing a thin coating of Ag_2_O NPs onto the surface of *Zea mays L.* with two ratios (e.g., 5 & 10%) to form novel cost-effective core-shell Ag₂O/*Z* nanostructures. To assess morphology, and elemental composition, the synthesized composite was examined using high-resolution transmission electron microscopy (HR-TEM) and scanning electron microscopy (SEM) together with energy dispersive X-ray (EDX) spectroscopy. The effectiveness of 10% Ag_2_O/Z as a catalyst and adsorbent was evaluated based on several criteria, including pH, beginning concentration of the target dye, and the amount of nanocomposite utilized. Significantly, 0.01 g of 10% Ag_2_O/Z attained a 95.0% elimination of MB at a solution pH of 9.0 after 120 min. Moreover, the Ag₂O NPs and Ag_2_O/Z core-shell nanostructures exhibited strong antibacterial effects, with the presence of Ag₂O NPs enhancing their antimicrobial properties and suggesting a synergistic effect with the *Zea mays L.* matrix against *Klebsiella pneumoniae (ATCC:10031)*,* Staphylococcus aureus (ATCC:13565)*,* Bacillus subtilis (DSM:1088)*,* and Candida albicans (ATCC:10231)*. In summary, Ag_2_O/Z core-shell nanostructure showed strong antibacterial activity against gram-positive, gram-negative, and fungal pathogens, as well as effective dye removal capabilities, making them a viable agent for industrial and environmental applications.

**Supplementary Information:**

The online version contains supplementary material available at 10.1038/s41598-025-22961-9.

## Introduction

 These days, one of the biggest environmental issues is water contamination. One of the major and fundamental contaminants in water pollution is dyes. The environment is seriously threatened by the increasing amount of wastewater containing dyes that is released from numerous human activities due to the rapid process of industrialized growth^1^. Numerous physicochemical methods have been used to get rid of wastewater, including membrane filtration, ozonation, flocculation, electrocoagulation, photocatalysis, and adsorption. The most efficient method for eliminating organic dyes from wastewater among these many purification techniques is thought to be liquid-phase adsorption^2^. Despite significant advances in antimicrobial therapies and dye-removal technologies, several drawbacks limit their effectiveness and sustainability. In the case of antimicrobial agents, the extensive use of antibiotics has led to the rapid emergence of resistant microbial strains, reducing treatment efficacy and increasing healthcare costs. Conventional antibiotics often exhibit limited stability, poor bioavailability, and can cause adverse side effects, while their overuse contributes to environmental contamination and persistence of resistant pathogens^3^. Similarly, existing dye-removal methods such as membrane filtration, coagulation–flocculation, and advanced oxidation processes face challenges including high operational cost, generation of toxic byproducts, complex maintenance, and low efficiency at high pollutant loads. Photocatalysts like TiO_2_, ZnO, and other metal oxides are effective, but their large-scale application is hindered by limited stability, high cost of synthesis, and in some cases, poor performance under variable environmental conditions. These limitations highlight the urgent need for developing sustainable, low-cost, and multifunctional materials that can simultaneously act as efficient photocatalysts for dye degradation and as antimicrobial agents against resistant pathogens^4^. Agricultural activities generate a variety of biomass waste, including *Zea mays L.*, sugar cane bagasse, tapioca onggok, and empty palm oil bunches. The potential for using this biomass as adsorbents is very high^5^. With an annual production of over 250 million tons and a variety of sources, *Zea mays L.* are an abundant agricultural residue on the domestic market that are reasonably priced^6,7^. Another method to lessen environmental effects, boost economic worth, and add value to the trash is to employ *Zea mays L.* biomass waste. The majority of *Zea mays L.* waste has not yet been utilized to its full potential and is typically turned into animal feed. Furthermore, the majority of *Zea mays L.* are burned for fuel, which seriously pollutes the environment and wastes a lot of resources^5^. Because waste biomass has advantageous properties including being modifiable, biocompatible, and biodegradable, it is being used as a source of renewable resources for a variety of applications as a result of current research and technical advancements^6,8^. *Zea mays L.* have a loose and porous structure, a large specific surface area, which endows them with adsorption capacity^9^. However, in general, natural *Zea mays L.* biomass has a poor adsorption capability. As a result, numerous modification techniques have been developed to boost their application in wastewater treatment^1^.

On the other hand, the utilization of silver oxide nanoparticles (NPs) has gained popularity in solving environmental issues, particularly in wastewater treatment^10–12^. However, it is very expensive, and this increases the cost of industrial application and hinders its use due to its low yield and high cost^13^. Therefore, it is essential to create simple, inexpensive, low-energy synthesis and modification technologies to optimize its production process^14^. Because of its simplicity, cleanliness, and ability to produce nanoparticles with specific qualities at a reasonable cost, the core shell technology is a more favorable method for preparing silver oxide nanoparticles than other approaches^15^. Every core/shell particle has a shell domain covering a core structural domain. The core and shell domains can be made up of a variety of materials, including metals, polymers, and inorganic solids. These particles’ mechanical, optical, magnetic, thermal, catalytic, electrical, and electro-optical properties can be easily modified by changing their size, composition, and structure. Coating the core particles has multiple advantages, such as making it easier to modify the surfaces of various wastes; improving the core particles’ functionality, stability, and dispersion; drastically lowering the need for costly materials; and allowing for controlled core release^15–21^.

The spread of infectious diseases poses a major threat to global public health, particularly when bacterial species that are resistant to antibiotics multiply. Recent advances in nanobiotechnology will probably lead to the creation of new antibacterial drugs. Nanomaterials’ remarkable physical, chemical, and biological properties have caught the interest of numerous businesses, including the biomedical sector^22^. Nanoparticles unique features that are used in a variety of disciplines, silver oxide nanoparticles (NPs) have a prominent position in nanomaterial research among the many nanoparticles that have been studied. Silver oxide nanoparticles’ antimicrobial qualities, which are frequently employed in antifungal and antibacterial applications, result from electrical changes that occur when they interact with bacterial membranes^23,24^.

*Moreover*,* Zea mays L.* biomass, primarily composed of several natural components such as cellulose, hemicellulose, and lignin, has garnered attention for its potential applications in antibacterial and photocatalytic processes. Research indicates that cellulose derivatives, when functionalized or combined with bioactive components, can exhibit antimicrobial properties^25^. Hemicellulose, particularly xylan, has shown the ability to inhibit the growth of various bacteria, including *Staphylococcus aureus* and *Escherichia coli*^26^. Lignin, known for its polyphenolic content, possesses inherent antibacterial and antiviral properties by damaging bacterial cell walls through electrostatic interactions^27^. Moreover, lignin-based nanoparticles have been developed to enhance antimicrobial activity significantly^28^. In photocatalytic applications, *Zea mays L.*-derived materials have demonstrated effectiveness; for instance, mesoporous carbon nanoparticles obtained from corncob waste have been utilized in TiO_2_ nanocomposites for dye degradation^29^. These findings underscore the multifaceted roles of *Zea mays L.* biomass components in promoting bacterial inhibition and facilitating photocatalytic processes, making them valuable in developing sustainable antibacterial agents and environmental remediation technologies.

The selection of silver-coated *Zea mays L.* nanostructures in this work is justified by several key advantages over existing commercial antibacterial and photocatalytic agents like Ag, TiO₂, ZnO, Cu, and MgO. Firstly, *Zea mays L.* is a sustainable agricultural waste product, promoting environmental sustainability by reducing waste and minimizing ecological impact, unlike many synthetic agents that rely on non-renewable resources. Additionally, *Zea mays L.* exhibits excellent biocompatibility, making it suitable for applications requiring contact with biological systems, such as medical devices. Furthermore, the unique porous structure of *Zea mays L.* enhances the performance of silver, providing effective antibacterial and photocatalytic properties. The key goal of the present work is to benefit from the *Zea mays L.* biomass waste and convert it to value-added material through chemical deposition of a thin layer of silver oxide nanoparticles (NPs) on its surface with two ratios (e.g., 5 & 10%) to form novel cost-effective core-shell nanostructures with good photocatalytic and antimicrobial activities.

## Materials and methods

### Materials

Silver nitrate of 99.9% purity was obtained from LOBA, India. Sodium hydroxide and Cetyl trimethyl ammonium bromide (CTAB) of 99% and 9.5% purity, respectively, were supplied from Adwic Co., Egypt. *Zea mays L.* was collected locally in Egypt. Methylene blue dye (MB, ≥ 97%) obtained from E-Merck Products. The experiment utilized analytical reagent grade chemicals, which were employed without any additional purification. The experiment utilized distilled water and deionized water for the preparation of solutions and for cleaning purposes.

### Synthesis of the nanoparticles

#### Synthesis of Ag_2_O NPs

Silver nitrate was stoichiometrically stirred vigorously for 1 h at room temperature with 2% (w/v) CTAB, which was used as a dispersing agent. Then, the deposition step was done using 2 N NaOH until pH 9. Finally, the mixtures were filtered, washed until pH 7, dried at 80 °C, and calcined at 500º^15,30^.

#### Synthesis of core/shell nanostructures

**Fig. 1:**
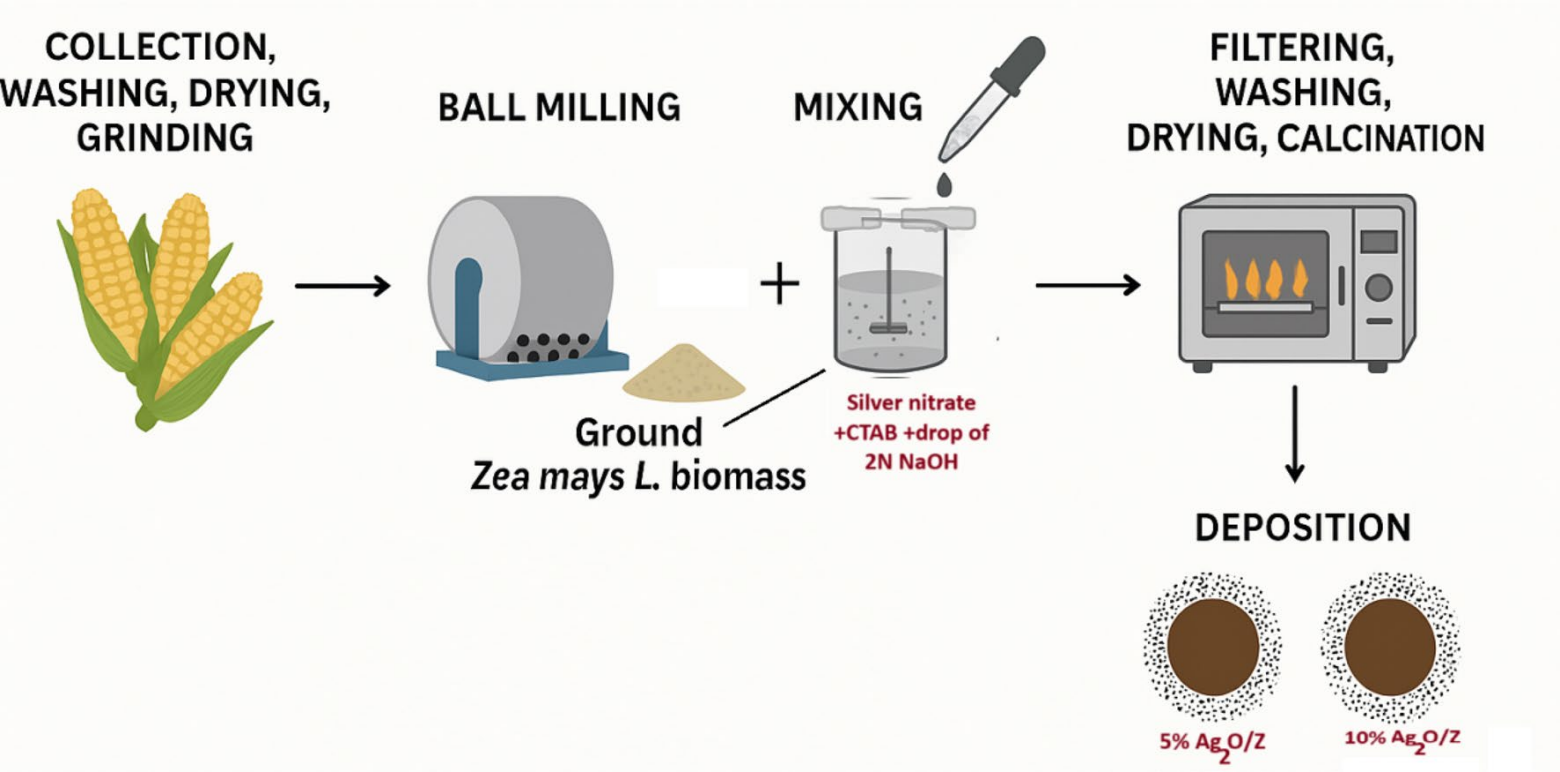
A schematic illustration of the synthesis process.

Firstly, *Zea mays L.* biomass waste was collected, washed, dried, and ground on the nanoscale using a ball mill. After that, silver nitrate was mixed stoichiometrically with two proportions (e.g., 5 & 10%) with 5 gm of the ground *Zea mays L.* biomass in the presence of 2% (w/v) CTAB under vigor stirring for 1 h. Then, the deposition step was done using 2 N NaOH until pH 9. Finally, the mixtures were filtered, washed until pH 7, dried at 80 °C, and calcined at 500°C^15,30^. The obtained core-shell nanostructures are 5% silver oxide/ *Zea mays L.* (5% Ag_2_O/Z) and 10% silver oxide/*Zea mays L.* (10% Ag_2_O/Z), as shown in Fig. 1.

### Methods of instrumental analysis

Transmission electron microscopy and scanning electron microscopy/energy-dispersive X-ray analysis techniques using micro-analyzer electron probes (JEOL JX 1230 and JEOL JX 2840) Japan, respectively were used to determine the shapes and sizes of *Zea mays L.* biomass and the prepared cores-shell nanostructures. The ZPW388 particle sizing system obtained from Santa Barbara, California, USA, was used. A JASCO FTIR-4100 E FT-IR spectrometer (Japan) operating in absorption mode in the wavenumber range of 4000 –400 cm^− 1^ was used to measure the FT-IR spectra of the synthesized cores-shell nanostructures.

### Photocatalytic degradation setup

**Fig. 2:**
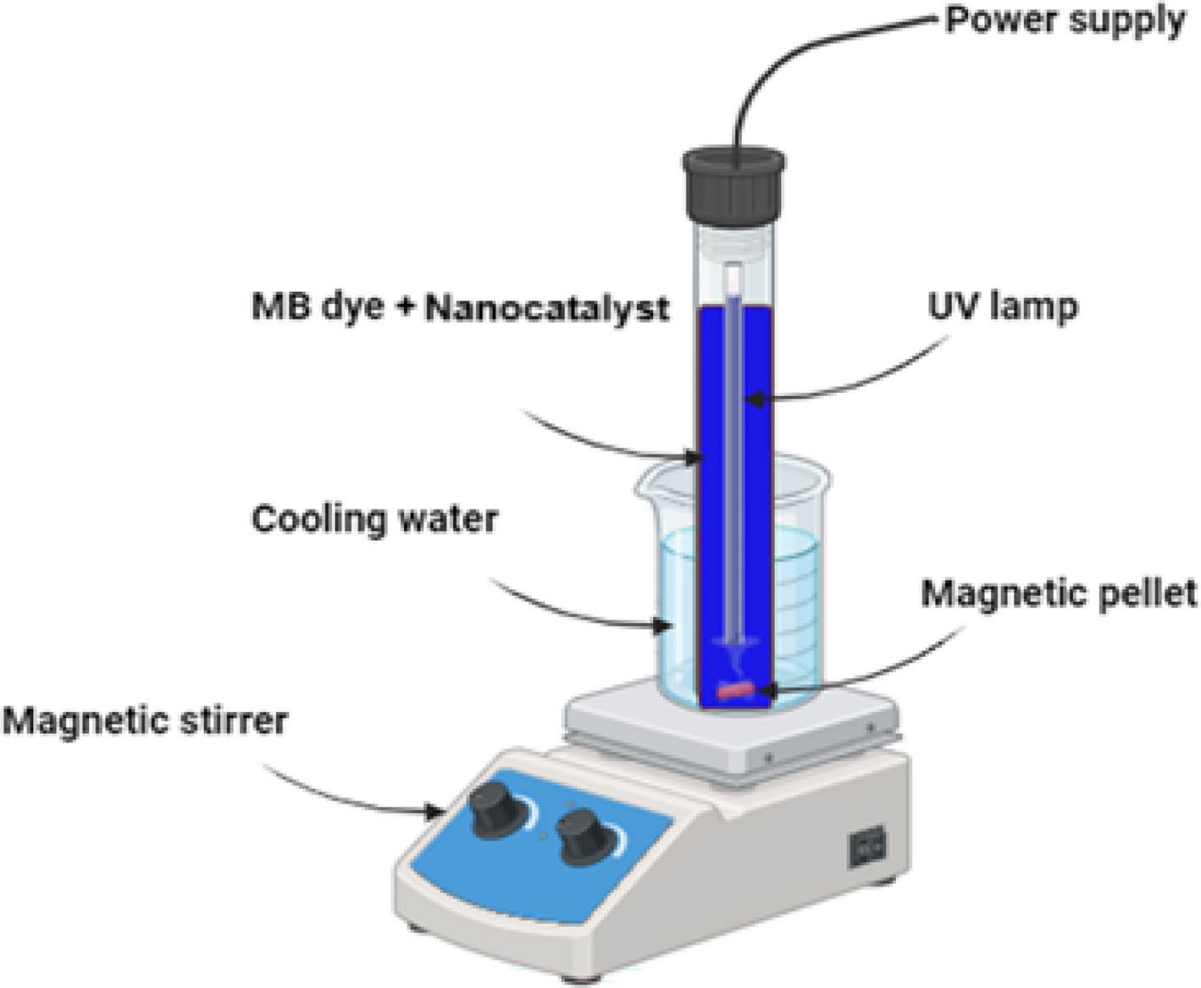
Schematic figure of the photocatalytic degradation setup of MB.

MB degradation was achieved using photocatalysis utilizing a UV lamp and silver oxide/*Zea mays L*. The existing UV reactor is a cylindrical glass vessel with a capacity of 100 ml with diameter of 3 cm, and a length of 27 cm. Aluminum foil is applied thinly to the outside of the reactor. There were 50 milliliters of contaminated solutions in the photoreactor. The ultraviolet light source used was the Philips TUV 11WG11 T5, a widely available UV-C lamp. With an output of 11 watts with ultraviolet radiation of 30.000 W/cm^2^, this high-pressure mercury lamp emits light with an average wavelength of 254 nm. The photoreactor is completely immersed in the tainted solutions, and a cold-water bath keeps it at a temperature of about 15 °C as illustrated in Fig. 2.

Initially, introduce the pollutant MB dye and the nanocatalyst into the glass cylindrical reactor. Subsequently, apply UV irradiation. Periodically extract a 2 ml suspension of the MB solution from the UV reactor with a syringe. Subject the suspension to centrifugal force for 20 min and measure the light absorption at a wavelength of 664 nm using a spectrophotometer^31^. Using the following formula, the photodecomposition efficiency (Removal %) was determined.:

1 $${\text{MB Removal }}\left( \% \right){\text{ }}=1 - \left( {\frac{{Ct}}{{C0}}} \right){\text{*}}100$$

Where Ct represents the concentration at a particular time (t), and Co is the starting concentration of MB in (mg/L). The initial pollutant concentrations and pH level were among the operating parameters of photocatalytic degradation that were investigated.

### Antimicrobial activity measurements

After filling sterile Petri dishes with 20–25 ml of nutritional agar and Sabouraud dextrose agar medium, the dishes were allowed to solidify at room temperature. Microbial suspensions were made in sterile saline to match the 0.5 McFarland standard solution (1.5 × 10^5^ CFU/ml), and a spectrophotometer set to 625 nm was used to adjust the turbidity to OD = 0.13^32^. A sterile cotton swab was dipped into the bacterial suspension and uniformly distributed throughout the agar surface within 15 min of the inoculum being adjusted. The plates were covered and allowed to dry for 15 min. Wells 6 mm in diameter were made in the solidified media using a sterile borer. Using a micropipette, a 100 µL aliquot of each tested compound solution was added to the wells. The antimicrobial activity was evaluated against representative Gram-negative, Gram-positive, and fungal strains. The tested microorganisms included *Klebsiella pneumoniae* (ATCC 10031), *Staphylococcus aureus* (ATCC 13565), *Bacillus subtilis* (DSM 1088), and *Candida albicans* (ATCC 10231). To test these microorganisms, plates were incubated for 24 h at 37 °C. The inhibitory zones were measured in millimeters, and this process was carried out in triplicate^33^.

### Statistical analysis

The experimental data were expressed using the calculated mean and standard deviation. The calculated mean is derived from triplicate data from three different experiments. With the use of IBM Corp.‘s SPSS software, version 22 (New York), one-way analysis of variance (ANOVA) and the least significant difference (LSD) test (0.05 level) were used to examine statistical significance.

## Results and discussion

### Characterization of the synthesized core-shell nanostructures

#### Morphological study using TEM

**Fig. 3:**
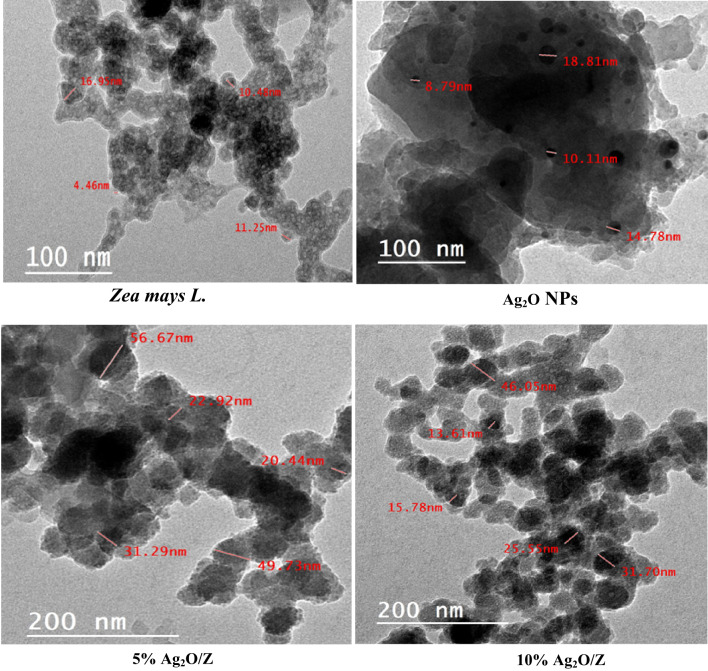
TEM images of *Zea mays L.* biomass and the synthesized core/shell nanostructures.

Figure 3 presents TEM images of both *Zea mays L.* biomass and the synthesized core/shell nanostructures. The images demonstrate that *Zea mays L.* biomass is extremely porous, nanoscale prticles with sizes varying from 4.46 to 16.95 nm. The images show that silver oxide nanoparticles (NPs) filled the surface of the porous particles of the *Zea mays L.* biomass in the case of synthesized core/shell nanostructures. In the image of 5% Ag_2_O/Z, the *Zea mays L.* biomass particles appeared surrounded and covered with silver oxide NPs with a percentage of less than 10% Ag_2_O/Z. Therefore, TEM images showed that Ag_2_O NPs were successfully deposited on the surface of *Zea mays L.* biomass.

#### SEM and EDX analysis

**Fig. 4:**
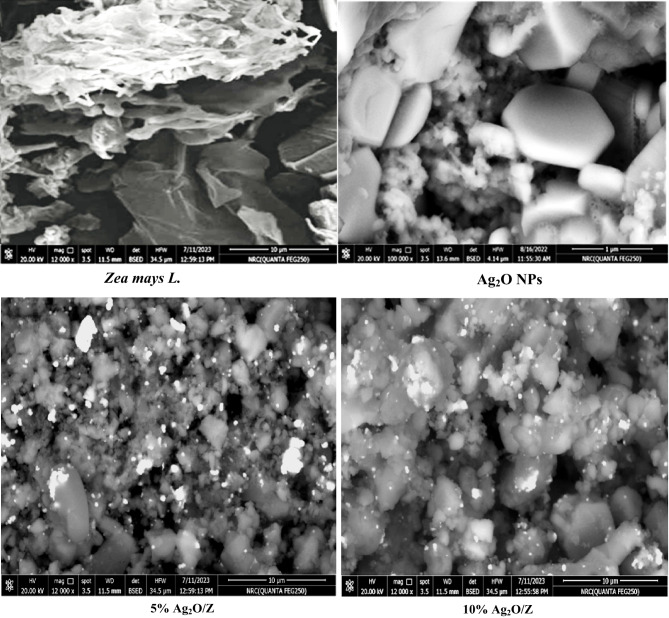
SEM images of *Zea mays L.* biomass and the synthesized core/shell nanostructures.

SEM for *Zea mays L.* biomass waste and the synthesized core/shell nanostructures is shown in Figure 4. *Zea mays L.* biomass’s SEM picture shows a porous, spongy surface structure; this high porosity can enhance the adsorption process^1^. Additionally, the generated core/shell nanostructures’ SEM micrographs revealed that silver oxide particles in the form of plates covered the surface of the spongy *Zea mays L*. biomass particles.

**Fig. 5:**
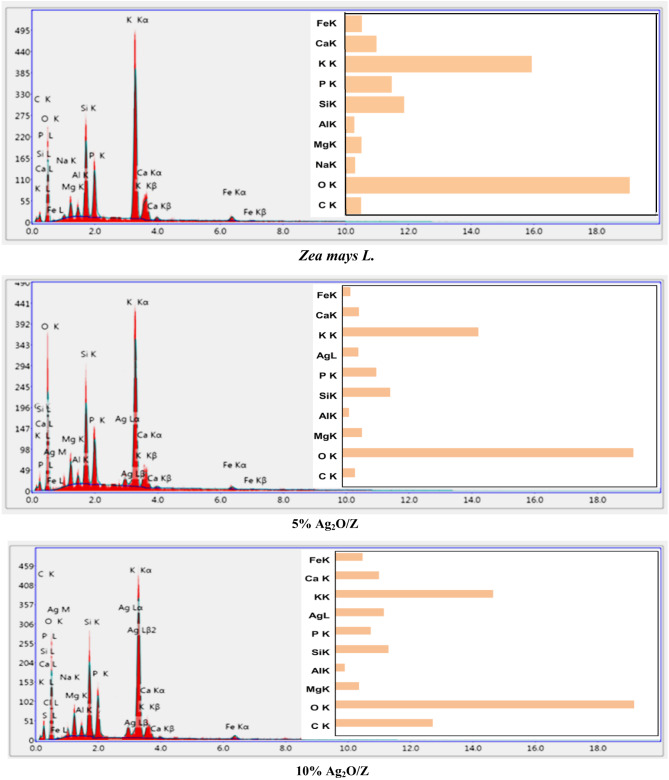
EDX analysis of the *Zea mays L.* biomass and the synthesized core/shell nanostructures.

Figure 5 presents the EDX analysis of *Zea mays L.* biomass waste and the synthesized core/shell nanostructures. Since this technique identifies surface elements to a depth of approximately one micron, it is commonly used to confirm the surface deposition of expensive nanomaterials onto more affordable substrates, such as *Zea mays L.* biomass waste.

EDX of *Zea mays L.* biomass waste indicates that it consists of many elements such as carbon, oxygen, silicon, sodium, potassium, magnesium, aluminum, phosphorus, calcium, and a very high percentage of potassium in addition to a small percentage of iron. EDX of the synthesized core/shell nanostructures indicates the presence of the silver element in addition to the appearance of the main elements present in the *Zea mays L.* biomass, and it also showed that the peak of the silver element was larger and clearer in 10% Ag_2_O/Z than in 5% Ag_2_O/Z.

#### Particle size distribution

**Fig. 6:**
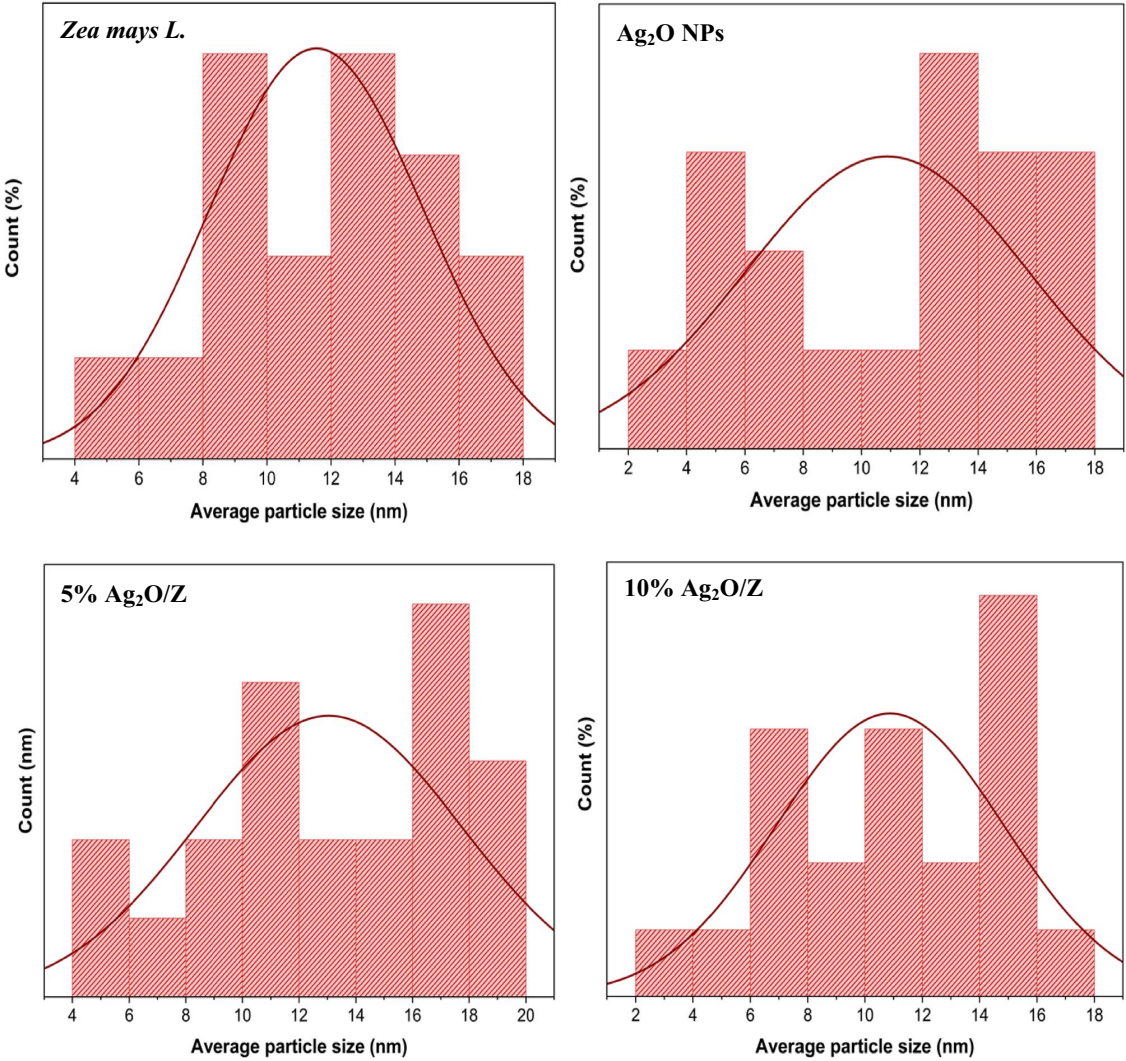
Particle size distribution of *Zea mays L.* biomass and the synthesized core/shell nanostructures.

Figure 6 elucidates the average particle size of *Zea mays L.* biomass and the core-shell nanostructures. It is found that the average particle size of *Zea mays L.* ranges from 4 to 18 nm and from 2 to 18 nm for Ag_2_O NPs. While in the case of the synthesized nanostructures, the average particle size ranges are 4–20 nm and 2–10 nm for 5% Ag_2_O/Z and 10% Ag_2_O/Z, respectively.

#### Zeta potential

**Fig. 7:**
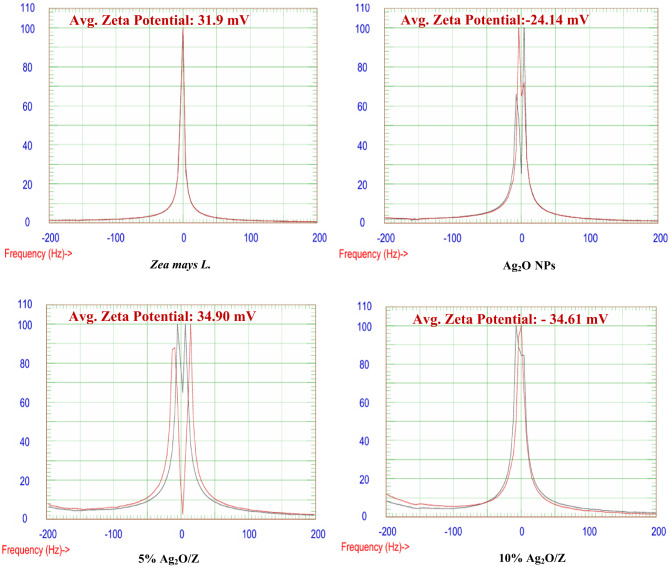
Zeta potential of *Zea mays L.* biomass and the synthesized core/shell nanostructures.

Figure 7 presents the zeta potential of *Zea mays L.* biomass and the core-shell nanostructures. Zeta potential is a critical parameter that indicates the stability of nanoparticles within their dispersive medium. Nanoparticles can achieve stabilization through electrostatics at zeta potential values exceeding ± 30 mV^34–37^. The figure shows that *Zea mays L.*, 5% Ag_2_O/Z, and 10% Ag_2_O/Z do not agglomerate and are within the required range, confirming their stability as their average zeta potential is 31.9, 34.9, and − 34.69 mV, respectively. In contrast, the average zeta potential of silver oxide NPS is less than ± 30 mV, as its zeta potential value is -24.14 mV. Therefore, the obtained results demonstrate that the deposition of silver oxide NPs on the surface of *Zea mays L.* has enhanced the dispersion of nanoparticles.

#### Fourier transform infrared spectroscopy

**Fig. 8:**
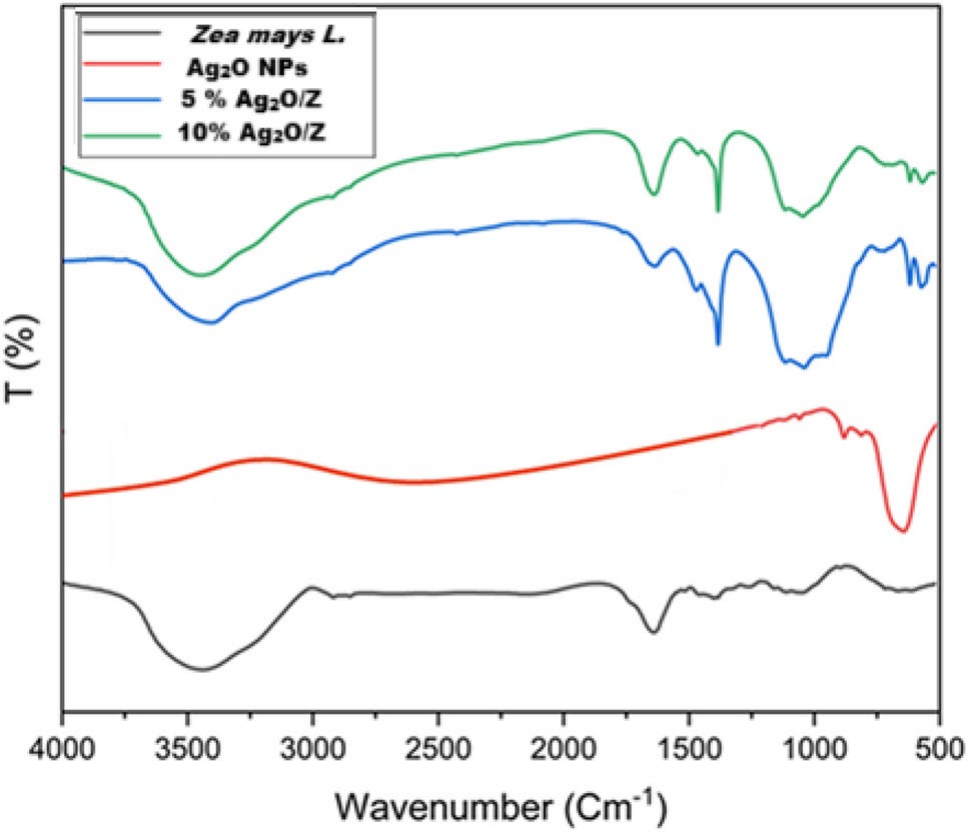
FTIR analysis of *Zea mays L.* biomass and the synthesized core/shell nanostructures.

Figure 8 illustrates the FTIR analysis of *Zea mays L. biomass* and various synthesized core/shell nanostructures. The spectrum for *Zea mays L.* shows characteristic peaks indicative of functional groups such as hydroxyl (–OH), methylene (–CH_2_), and carbonyl (C = O), with notable peaks around 3300 cm^–1^ for –OH stretching and 800–1200 cm^–1^ for C–O stretching in cellulose and hemicellulose. Furthermore, the region between 1190 and 1220 cm^–1^ is assigned to the stretching vibrations of C–O, C–N, and C–S bonds.

The absorption near 2920 cm^–1^ is associated with C–H stretching vibrations of alkyl groups. Peaks observed between 1620 and 1690 cm⁻¹ are indicative of aromatic and olefinic C = C stretching^28^. The spectrum for silver oxide nanoparticles (Ag_2_O) displays distinct peaks, indicating modifications in chemical structure due to synthesis. For Ag₂O nanoparticles, a prominent peak observed around 550 cm^–1^ corresponds to the stretching vibration of the Ag–O bond^38^. The 5% Ag_2_O/Z nanostructure spectrum reveals deposition of silver oxide nanoparticles on the surface of *Zea mays L.*, with shifts in peak positions and intensities compared to the *Zea mays L.* spectrum. This trend continues in the 10% Ag_2_O/Z spectrum, where further modifications suggest enhanced interactions due to a higher silver concentration. Overall, the FTIR analysis highlights the successful deposition of silver oxide nanoparticles into the surface of *Zea mays L.*, providing insights into the material’s chemical composition and its potential applications in antibacterial and photocatalytic fields.

### Photocatalytic degradation of MB dye using 10% Ag_2_O/C NPs

**Fig. 9:**
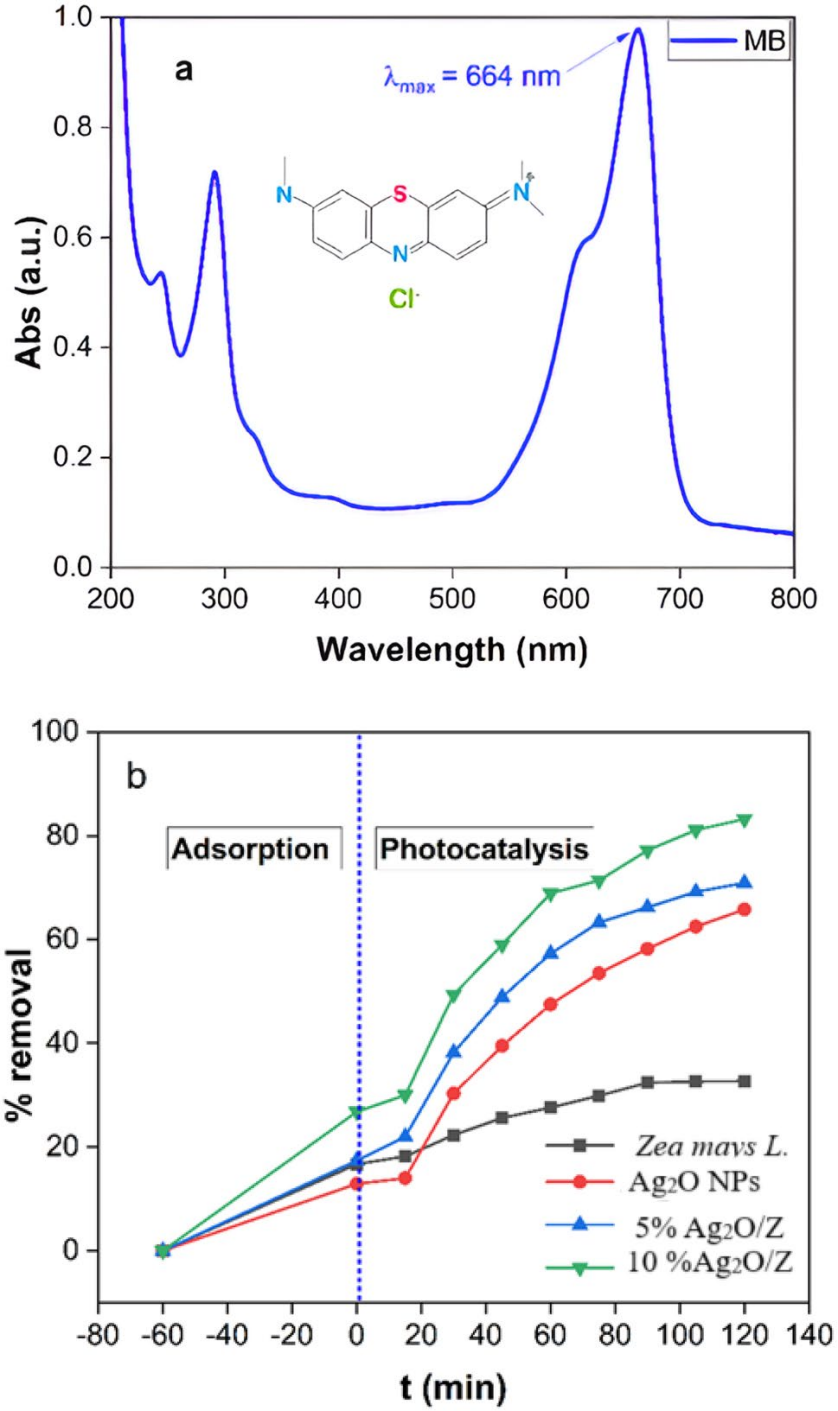
**(a)** UV-Vis spectrum of MB (λmax = 664 nm), **(b)** photocatalytic reduction of MB by *Zea mays L.*, Ag_2_O NPs, 5% Ag_2_O/Z NPs and 10% Ag_2_O/Z NPs under UV irradiation.

The removal of MB was tracked using spectrophotometry at the absorbance peak of the MB dye, which is λmax = 664 nm, as depicted in Fig. 9^9^^31,39^.

Figure 9b indicates that the adsorption of MB in the absence of light resulted in a clearance rate of 29.0% during a duration of 60 min. The findings indicate that the 10% Ag_2_O/Z NPs sample markedly improves the removal efficiency of MB to around 85.0% after 120 min at pH 7.0, in contrast to bare *Zea mays L.*, Ag_2_O NPs, and 5% Ag_2_O/Z NPs, which exhibit efficiencies of 32.4%, 65.3%, and 72.5%, respectively.

In accordance with established literature^31,40^, methylene blue exhibited high photostability, with minimal degradation observed upon direct UV irradiation; this confirms that the generation of reactive radicals via photolysis alone is ineffective for MB decomposition in the absence of a photocatalytic agent. The improved photocatalytic activity can be attributed to the formation of a metal–semiconductor heterojunction within the nanoparticles, which facilitates more efficient charge separation and enhances light absorption. Also, Metal-Semiconductor Heterojunction refers to the interface formed between a metal (Ag₂O) and a semiconductor (the carbon matrix from *Zea mays L.*). The junction between these two materials plays a critical role in the photocatalytic process. On the other hand, when light is absorbed by the photocatalyst, it generates electron-hole pairs. In a heterojunction, the arrangement of energy levels allows for more efficient separation of these charges. The electrons can be transferred to the metal (Ag₂O), while the holes remain in the semiconductor. This separation reduces the likelihood of recombination, where electrons and holes would otherwise cancel each other out. So, the formation of the metal-semiconductor heterojunction in the 10% Ag_2_O/Z NPs enhances photocatalytic activity by promoting efficient charge separation and improving light absorption, leading to more effective photocatalytic degradation of MB^41^.

#### Effect of pH and point of zero charge on removal of MB

**Fig. 10:**
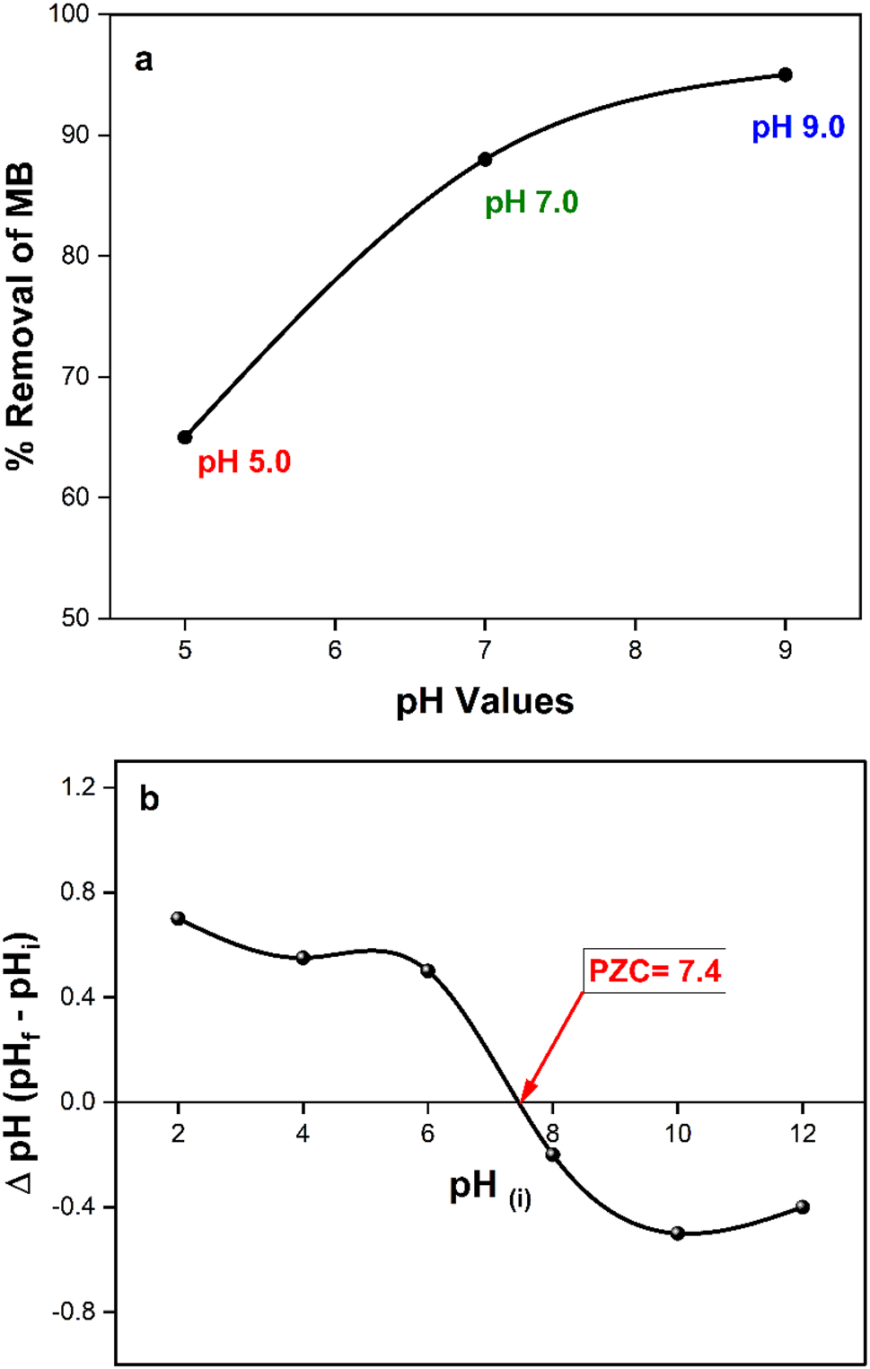
**(a)** Variation in methylene blue (MB) removal efficiency (%) over time at different solution pH levels (5.0, 7.0, and 9.0), using 10 mg of 10% Ag_2_O/Z NPs in 50 mL of 10 mg/L MB solution at 25 °C. **(b)** Determination of the point of zero charge (PZC) of 10% Ag_2_O/Z NPs across various pH values.

The solution’s pH dependency must be considered when doing removal investigations. To determine how various starting pH values affected the MB solution, an investigation was conducted. The study utilized 10 mg of 10% Ag_2_O/Z nanoparticles, 50 ml of a 10 mg/L methylene blue solution, and a temperature of 25 °C. The investigation lasted 120 min. A graph showing the percentage of MB elimination over time for three different solution pH levels (5.0, 7.0, and 9.0) is shown in Figure 10a. The maximum amount of MB was removed at equilibrium at a pH of 9.0. To determine the 10% Ag_2_O/Z nanoparticles’ point of zero charge (PZC), 0.01 g of the particles were added to a 50 ml solution that included 0.01 moles of NaCl. Either HCl or NaOH was added to the solutions to change their pH, resulting in values of 2, 4, 6, 8, 10, and 12. For 48 h, the specimens were agitated at a rate of 200 rotations per minute^42^. After 10% Ag_2_O/Z nanoparticles were separated magnetically, the pH levels of the solutions were determined.

By graphing the initial pH against the pH change (initial pH - final pH), the point of zero charge (PZC) was determined. Figure 10a illustrates the exhibited results. The pH value at which the final and initial pH values are identical is known as the point of zero charge (PZC), as shown in Figure 10b. The PZC exhibited a pH value of 7.4. The photocatalyst 10% Ag_2_O/Z NPs demonstrates a positive surface charge at pH levels below the point of zero charge (PZC) and a negative surface charge at pH levels above the PZC. The discovery elucidates the observed peak degradation of MB via photocatalysis at pH 9.0, as illustrated in Figure 10a. As a result, the 10% Ag_2_O/Z nanoparticles have a negative net surface charge, which attracts the positive charge of methylene blue electrostatically. The photocatalytic degradation of methylene blue (MB) is improved by this catalyst. At a pH of 5.0, the positive charge of MB and the total positive surface charge of the 10% Ag_2_O/Z NPs created repulsive forces.

On the other hand, at higher pH levels, the solubility of silver oxide in (Ag₂O/Z) NPs can increase, leading to a higher concentration of reactive silver species in the solution. This enhanced availability of silver ions could promote more efficient photocatalytic activity, facilitating the generation of reactive radicals necessary for dye degradation^43^. Also, the behavior of MB itself can be influenced by pH. At pH 9.0, the deprotonation of MB may reduce its tendency to aggregate, resulting in a more effective interaction with the catalyst surface. This increased dispersion can enhance the accessibility of MB to the reactive sites on the Ag₂O/Z NPs, improving degradation efficiency^44^.

#### Effect of initial concentration of MB and nanocatalyst dose on photocatalytic reaction

**Fig. 11:**
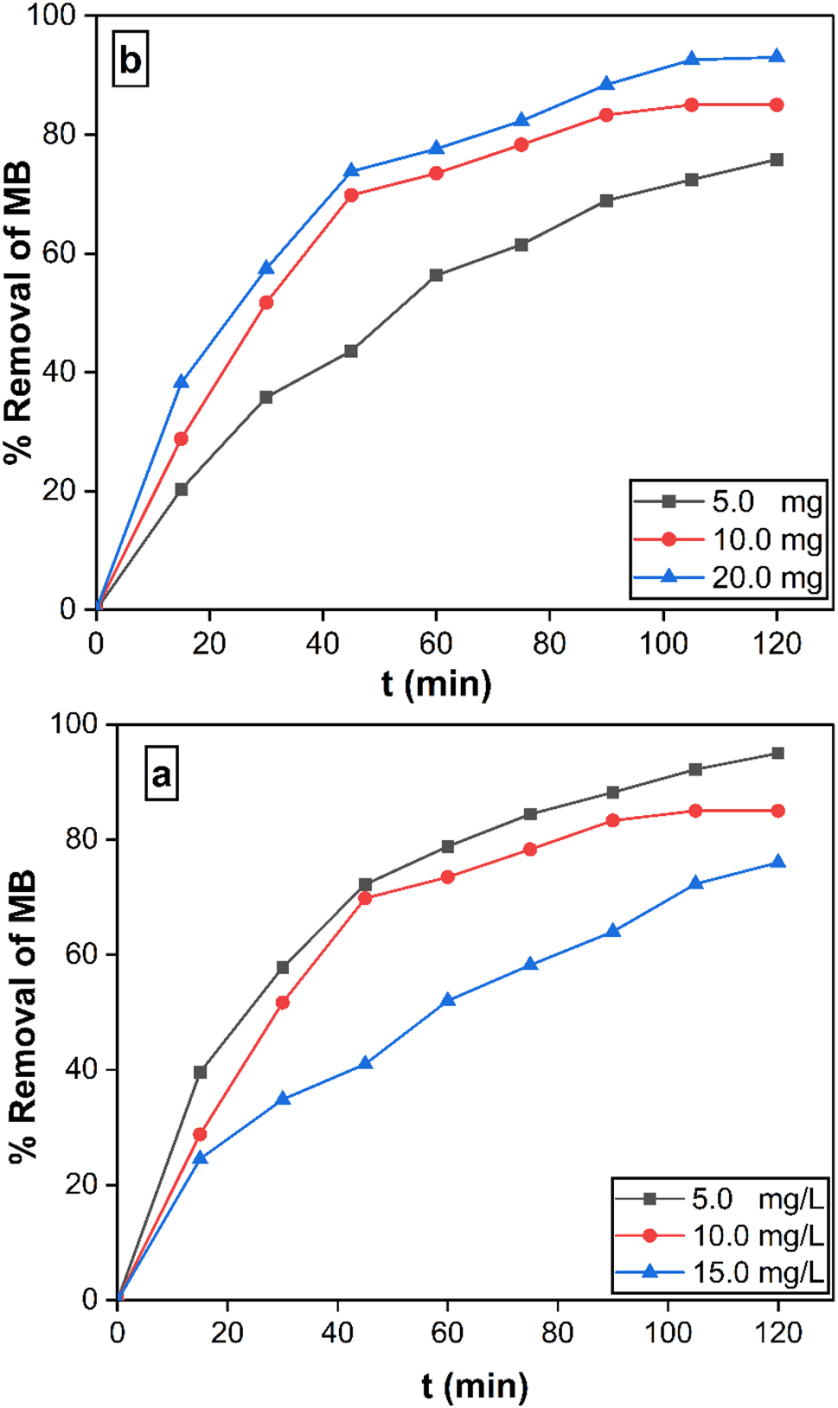
**(a)** Variation in the percentage of methylene blue (MB) removal as a function of contact time at different initial MB concentrations (5–15 mg/L), using 10.0 mg of 10% Ag₂O/Z NPs at pH 7.0. **(b)** Effect of photocatalyst dosage on the removal efficiency of MB from 50 mL solutions with concentrations ranging from 5.0 to 20.0 mg/L at 25 °C and pH 7.0.

In order to investigate the impact of ionic strength on MB, its initial concentration was varied while all other reaction parameters remained constant^31^. The elimination mechanism depends critically on the initial MB concentration. The correlation between the duration of contact and the percentage of elimination for different starting MB concentrations (5.0, 10.0, and 15.0 mg) is shown in Fig. 11a. The decreasing effectiveness of the generated 10% Ag_2_O/Z NPs nanoparticles in lowering the methylene blue concentration at different starting concentrations is shown in Figure 10a. The results show that when MB concentrations rise, the degradation efficiency falls. However, when exposed to UV light, the nanocomposite effectively eliminates MB, even at high initial concentrations. A study was carried out to assess how various nanoparticle dosages affected the effectiveness of methylene blue elimination when exposed to UV light. While the concentration of MB was maintained at 10 mg/L, the amount of photocatalyst employed ranged from 5 to 20 mg. The results are presented in Fig. 11b. The findings indicated that increasing the photocatalyst dosage from 5 to 20 mg resulted in a considerable improvement in removal efficiency. The improvement in removal efficiency reported with increased photocatalyst amount in the reaction may be explained by an increase in the photocatalyst’s accessible active area or active sites in respect to the volume of the MB solution^45,46^.

#### Kinetic studies

The following formula was used to calculate the MB removal rate:

2 $$- ln~Ct/C0~=~ - ~Kt~$$

**Fig. 12:**
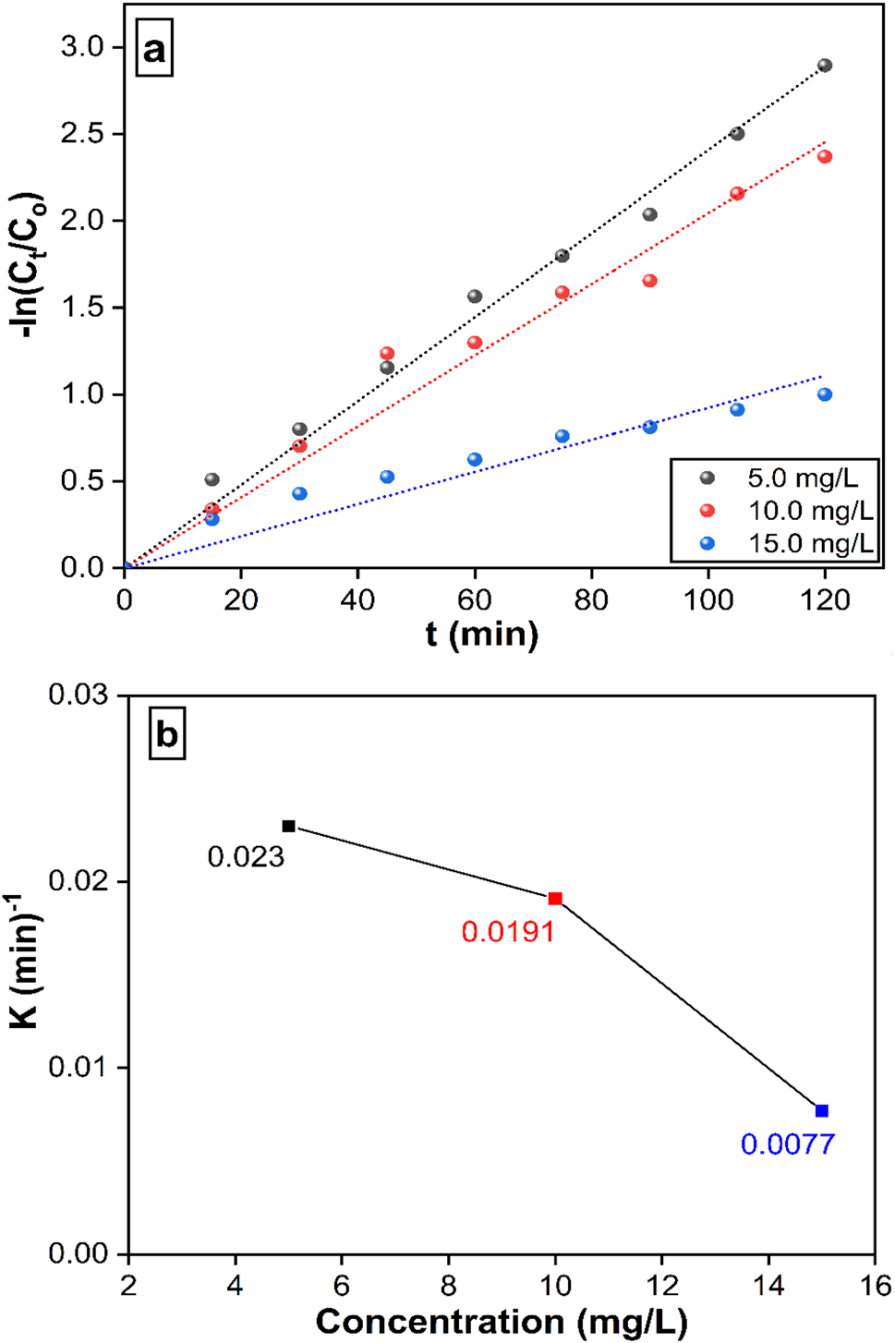
**(a)** Linear fitting of the kinetic data using the pseudo-first-order model for methylene blue (MB) degradation under UV-light irradiation at initial MB concentrations of 5, 10, and 15 mg/L. **(b)** Relationship between the apparent rate constants (k) from the pseudo-first-order model and the initial MB concentrations.

Where C_t_ represents the residual concentration of MB, whereas C_o_ represents the initial concentration of MB. The duration for removal is denoted as (t), whilst the removal rate constant is shown as (k). Figure 12a illustrates the correlation between the natural logarithm of the ratio of C_t_ to C_o_ and the variable t. The findings demonstrate that the elimination rate adhered to a pseudo-first-order model. An increase in the initial concentration of MB led to a corresponding increase in the apparent pseudo-first-order rate constants. Figure 12b. The relationship between MB concentration and reaction rate constants is consistent with previous work^47^.

#### Reuse and recycling

**Fig. 13:**
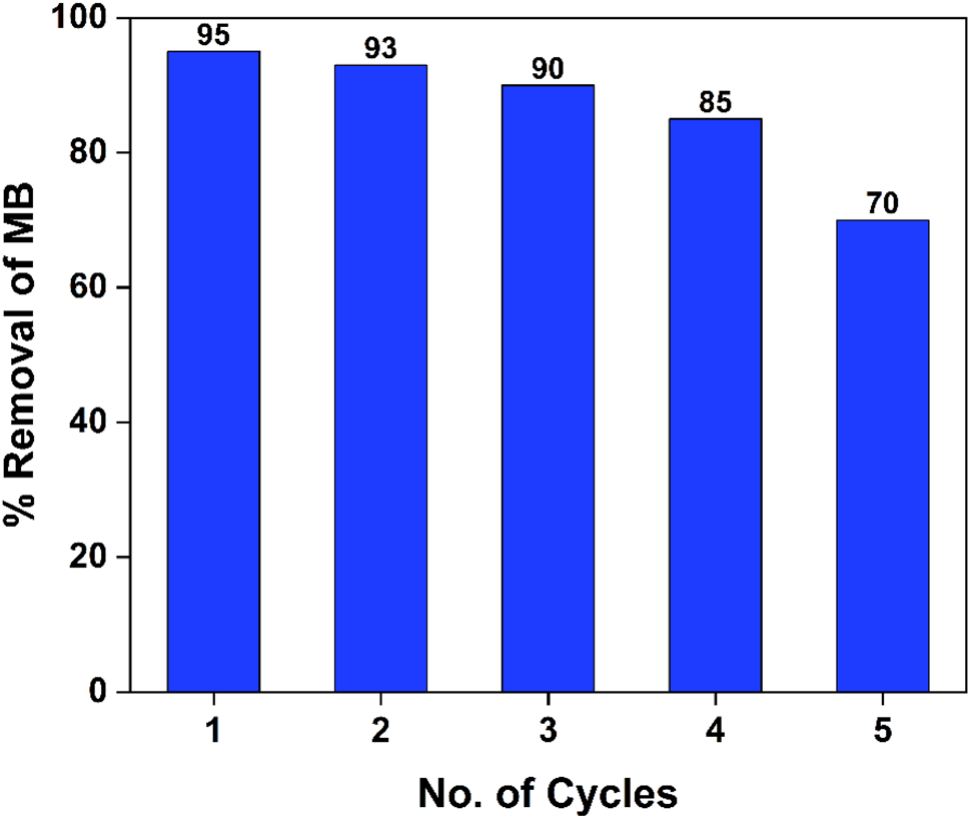
Recyclability of 10% Ag_2_O/Z NPs for MB degradation under UV light irradiation.

Over time, waste management requires the extraction and reuse of photocatalysts employed in environmental remediation^48^. Consequently, the 10% Ag_2_O/Z NPs sample demonstrated enhanced photocatalytic degradation efficiency and cost-effectiveness. Photocatalyst stability and long-term photocatalytic activity are both critical features. Reuse stability of a photocatalyst is essential to its industrial applications. The photocatalyst was collected by magnet, cleaned thrice with deionized water then let dry in an oven for eight hours at 60 °C before being used in the next cycle. Further research was carried out, as indicated in Fig. 13, to examine the reusability of 10% Ag_2_O/Z NPs in the photocatalytic reduction of MB dye under UV light irradiation. The photocatalytic reusability was conducted in the same manner as for the assessment of photocatalytic activity previously described. The photocatalytic activity of 10% Ag_2_O/Z NPs reduced to 70% after 5 cycles.

#### Mechanism of MB photocatalysis

The photodegradation mechanism, which is responsive to pH variations, may play a role in the potential process. In this mechanism, hydroxyl radicals are reduced by the conduction band’s electrons and oxidized by the valence band’s positive holes. The formation of electron-hole pairs on the photocatalyst’s surface upon exposure to ultraviolet light is a possible mechanism for photocatalytic degradation^49,50^.

Hydroxyl radicals and degradation products can be produced by the oxidative potential of holes, contingent upon their reaction with -OH groups or the oxidation of reactive MB. This is a concise summary of the mechanisms involved in the photocatalytic breakdown of methylene blue (MB): (3–6).

3 $${\text{1}}0\% {\text{ A}}{{\text{g}}_{\text{2}}}{\text{O}}/{\text{Z NPs}}+{\text{ h}}\nu ~~~~~~ \to ~~~~~{\text{1}}0\% {\text{ A}}{{\text{g}}_{\text{2}}}{\text{O}}/{\text{Z NPs}}\left( {{{\text{e}}^ - }_{{{\text{CB}}}}+{\text{ }}{{\text{h}}^+}_{{{\text{VB}}}}} \right)$$

4 $${{\text{h}}^+}_{{{\text{VB}}}}+{\text{1}}0\% {\text{ A}}{{\text{g}}_{\text{2}}}{\text{O}}/{\text{ZNPs}}~ \to {\text{1}}0\% {\text{ A}}{{\text{g}}_{\text{2}}}{\text{O}}/{\text{ZNP}}{{\text{s}}^+}\left( {{\text{Oxidation of the compound}}} \right)$$

Or

5 $${{\text{h}}^+}_{{{\text{VB}}}}+{\text{ O}}{{\text{H}}^{ - ~}} \to {\text{O}}{{\text{H}}^.}$$

6 $${\text{O}}{{\text{H}}^.}+{\text{ MB}}~~ \to ~~~~~~~~~~~\left( {{\text{Degradation products}}} \right)$$

**Fig. 14:**
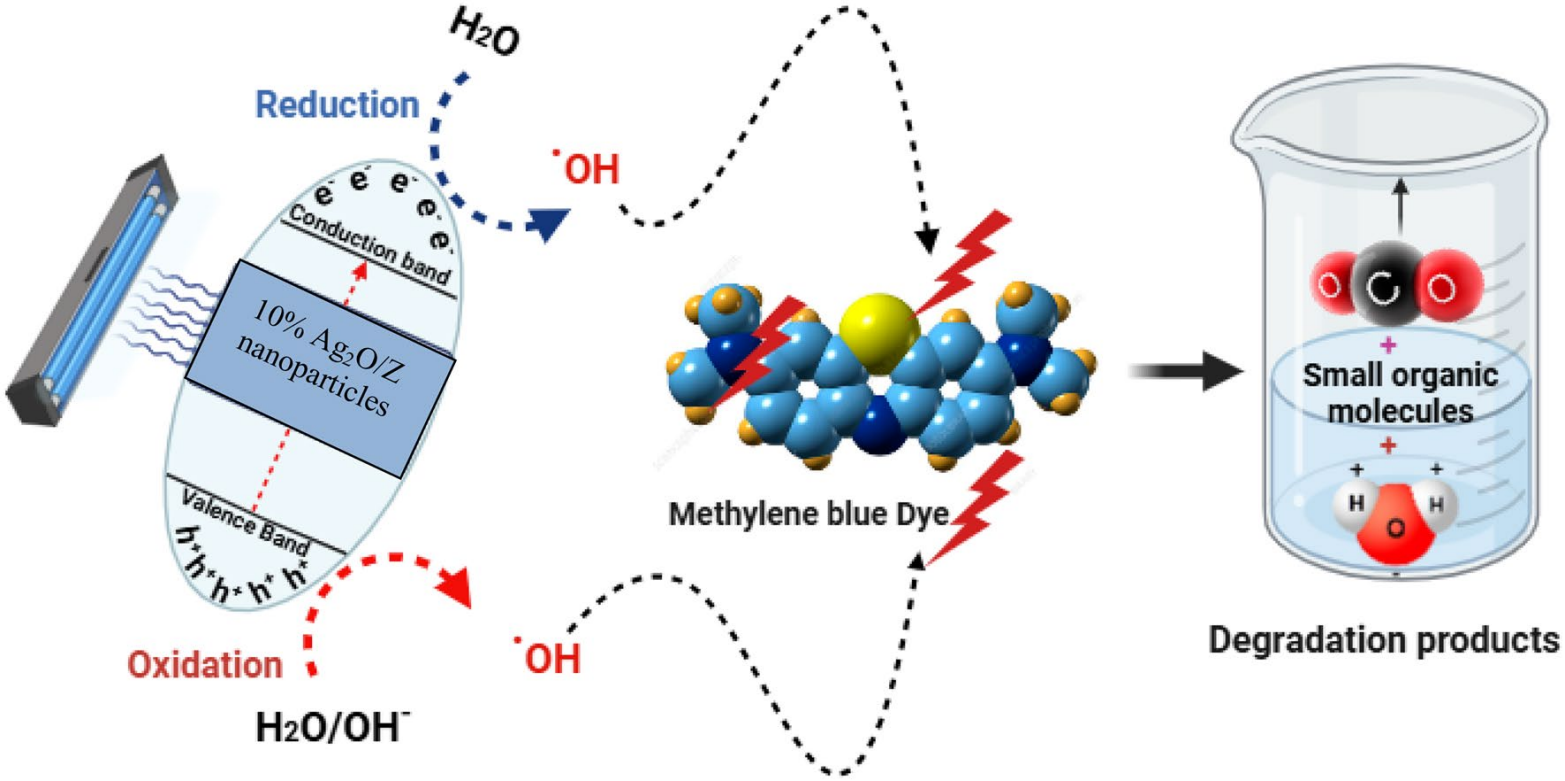
The possible photocatalytic reaction mechanism for MB photodegradation by 10% Ag_2_O/Z NPs.

Figure 14 depicts the proposed process elucidating the interaction between 10% Ag_2_O/Z nanoparticles and methylene blue. The absorption of photons by 10% Ag_2_O/Z nanoparticles under visible light can trigger redox processes through the generation of charge carriers. Therefore, the generated free radicals, such as OH· and O_2_·−, facilitate the breakdown of MB and lead to production of simpler organic molecules.

**Fig. 15:**
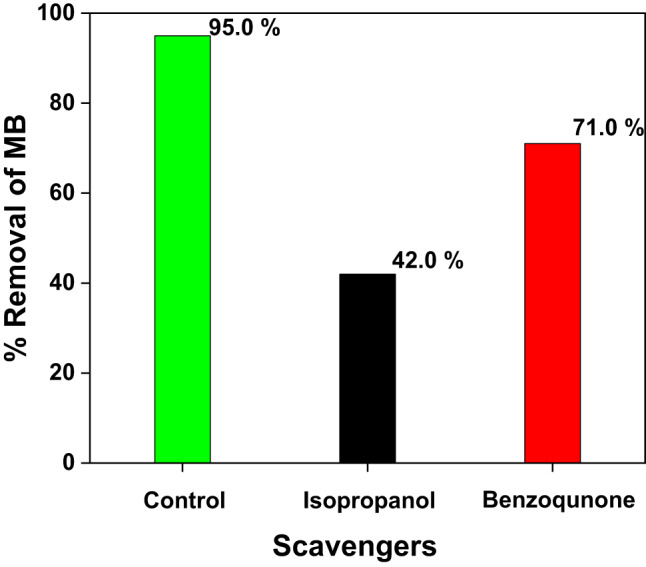
Effect of scavengers on MB photodegradation by (10% Ag_2_O/Z) NCs.

The most active species were identified by examining the impact of scavengers on the photocatalytic reduction process. The isopropanol and benzoquinone were used to capture OH· and − O2·, respectively^46^. 10% Ag_2_O/Z nanocomposite’s photocatalytic degradation of MB under UV irradiation is illustrated in Fig. 15, which illustrates the efficiency of this process in the absence and presence of scavengers at a concentration of 5 ppm. Efficiency decreased from 95% to approximately 71% and 42% in the presence of benzoquinone and isopropanol, respectively. The removal of MB by the 10% Ag_2_O/Z was reduced to (42%) when isopropanol was added, indicating that the OH· radical was the primary species responsible for MB degradation. In addition, the degradation rate was reduced by 71% by the addition of benzoquinone, which indicates that the − O2· radical played a significant role in the degradation of MB^51^. In addition, the comparison between this work and other photocatalysts for photocatalytic degradation of MB is shown in Table 1.

**Table 1:** Different photocatalysts for the photocatalytic degradation of MB.

Photocatalyst	Radiation/Light source	Degradation activity %	Time (min)	Ref.
Ag-ZnO	Visible light	93	80	^52^
ZnO	UV light	69	200	^53^
MgO	UV light	75	120	^54^
Ag2O/MgO/GO	Visible light	65	60	^55^
Co/TiO2	UV light	62	150	^56^
Ag-ZnO (0–6%)	Visible light	98	120	^57^
Ag-doped ZnO (0–1%)	Sollar light	98	120	^58^
10% Ag_2_O/Z	UV light	95	120	This study

### Antibacterial activity measurements

The agar well diffusion method was used to evaluate the investigated drugs’ antibacterial activity. Using nutritional agar medium, antibacterial activity was assessed against Gram-positive bacteria, such as *Staphylococcus aureus* (ATCC:13565) and *Bacilus subtilis* (DSM:1088), as well as Gram-negative bacteria, such as *Klebsiella pneumoniae* (ATCC:10031). Additionally, Sabouraud dextrose agar medium was used to assess the antifungal ability against *Candida albicans* (ATCC:10231). The standard antibiotics for Gram-positive and Gram-negative bacteria were ampicillin and gentamicin, respectively, and the standard antifungal was nystatin. As the negative control, (10% DMSO) was used. As indicated in Table 2, the compounds were tested against strains of bacteria and fungi at a dosage of 15 mg/ml.

**Table 2:** Antibacterial and antifungal activity of Ag₂O and Ag₂O/Z nanocomposites against selected microbial strains, measured as Inhibition zone diameters (mm). Standard antibiotics were used as positive controls.

Strains	Sample					
	*Zea mays L.* (ZOI/mm)	Ag_2_O (ZOI/mm)	5% Ag_2_O/Z (ZOI/mm)	10% Ag_2_O/Z (ZOI/mm)	10% DMSO	Standard antibiotic
Gram negative bacteria						Gentamicin
*Klebsiella pneumoniae* *(ATCC:10031)*	9.0 ± 1.0	24.7 ± 0.6	15.0 ± 1.0	12.7 ± 0.6	NA	25.3 ± 0.6
Gram positive bacteria						Ampicillin
*Staphylococcus aureus* *(ATCC:13565)*	NA	16.7 ± 0.6	18.7 ± 0.6	17.7 ± 0.6	NA	21.3 ± 0.6
*Bacilus Subtits* *(DSM:1088)*	NA	14.7 ± 0.6	14.0 ± 1.0	13.7 ± 0.6	NA	21.7 ± 0.6
Fungi						Nystatin
*Candida albicans* *(ATCC:10231)*	NA	15.0 ± 1.0	12.3 ± 0.6	11.0 ± 1.0	NA	21.7 ± 0.6

Data are presented as mean ± standard deviation (*n* = 3).

- Zone of inhibition is expressed as Mean ± Standard Deviation (mm).
- NA: No activity detected.
- Well diameter: 6 mm.
- 100 µl of each sample was tested.

The antibacterial activity of Ag_2_O and Ag_2_O/ *Z* nanocomposites varied depending on the microbial strain. Ag₂O exhibited the highest inhibition zones, particularly against *Klebsiella pneumoniae* (24.7 ± 0.6 mm), compared to the Ag₂O/ *Z* nanocomposites as represented in Fig. S1. The 5% and 10% Ag_2_O/ *Z* nanocomposites showed reduced antibacterial activity but remained effective, especially against *Staphylococcus aureus* and *Bacillus subtilis*. In terms of antifungal activity, both composites displayed moderate inhibition against *Candida albicans*, with a slight decrease in activity as Ag_2_O concentration decreased. These findings suggest that Ag₂O is the primary antimicrobial agent, while the *Zea mays L.* matrix may influence its effectiveness.

Interestingly, pure Ag_2_O exhibited stronger inhibition against Gram-negative *K. pneumoniae* (24.7 ± 0.6 mm) compared to the 10% Ag_2_O/Z composite (12.7 ± 0.6 mm), whereas the composite performed comparably or better against Gram-positive *S. aureus* (17.7 ± 0.6 mm vs. 16.7 ± 0.6 mm for pure Ag_2_O). These differences may be attributed to variations in cell wall structure between Gram-negative and Gram-positive bacteria. In Gram-negative species, the outer membrane acts as an additional barrier, potentially limiting the release or diffusion of Ag^+^ ions from the composite. By contrast, Gram-positive bacteria with thicker but more permeable peptidoglycan layers may allow greater interaction of the Ag_2_O/Z matrix with the cell wall, enhancing antimicrobial efficacy. Thus, the *Zea mays L.* matrix may contribute synergistically to certain bacterial contexts, particularly against Gram-positive strains.

**Table 3:** Antibacterial applications of some coated metal oxide nanoparticles against gram positive and gram negative bacteria.

Metal oxide nanoparticle	Coat	Preparation method	Size (nm)	Pathogen	ZOI	Ref.
Iron oxide nanoparticles (Fe_3_O_4_ NPs)	Chitosan	Co-precipitation	21.0	*E.coli*	18.0 ± 0.5	^59^
				*S. aureus*	10.0 ± 0.5	
CoFe_2_O_4_ nanoparticles	Capsaicin	Co-precipitation	25.0–35.0	*E.coli*	17.0	^60^
				*S. aureus*	23.5	
Ag NPs	Amoxicillin	Chemical reduction method	**35.5**	*E.coli*	40 ± 1.2	^61^
Fe_3_O_4_ nanoparticles	*Cinnamomum tamala* (CT) leaves	Co-precipitation	26–35	*E.coli*	7.0	^62^
				*S. aureus*	8.0	
	*Jatropha curcas* (JC)		20–42	*E.coli*	7.0	
				*S. aureus*	6.5	
Ag_2_O nanoparticles	*10% Zea mays L.*	Chemical deposition	2.0–10.0	*Klebsiella pneumoniae*	12.7 ± 0.6	This Study
				*S. aureus*	17.7 ± 0.6	

Statistical analysis (one-way ANOVA, Duncan’s multiple range test, *p* < 0.05) was applied to inhibition zone data to assess differences between treatments. While Ag_2_O generally showed the strongest inhibition, 5% and 10% Ag_2_O/Z composites exhibited statistically comparable or enhanced activity against *S. aureus* (*p* < 0.05), suggesting possible synergistic effects of the *Zea mays L.* matrix. No significant differences were observed between Ag_2_O and Ag_2_O/Z against *K. pneumoniae*, indicating that Ag₂O alone contributed more strongly to activity in Gram-negative bacteria. In addition, the comparison between this work and other metal oxide nanocomposites coated with polymers and natural products for antibacterial applications is shown in Table 3.

### Proposed antimicrobial activity mechanism of Ag_2_O NPs

The antimicrobial action of Ag₂O nanoparticles (NPs) is primarily mediated through two synergistic pathways: reactive oxygen species (ROS) generation and Ag^+^ ion release (leaching). Upon exposure to aqueous environments, Ag_2_O NPs partially dissolve, releasing Ag^+^ ions. These ions have a high affinity for thiol (-SH) groups in proteins, leading to the inactivation of vital bacterial enzymes involved in respiration and DNA replication. Ag^+^ ions can also intercalate with bacterial DNA, disrupting the helical structure and inhibiting replication^63^.

Simultaneously, photoexcitation or redox reactions at the Ag_2_O surface promote the generation of ROS, such as hydroxyl radicals (·OH), superoxide anions (O_2_^·–^), and singlet oxygen (^1O_2_). These ROS attack bacterial membranes through lipid peroxidation, leading to increased permeability and eventual lysis. They also oxidize nucleic acids and proteins, creating oxidative stress that overwhelms bacterial defense systems. The combination of direct Ag⁺ toxicity and ROS-induced oxidative stress results in irreversible damage to bacterial cellular machinery^64^.

At the molecular level, Ag_2_O NPs attach electrostatically to the negatively charged bacterial cell wall components (lipopolysaccharides in Gram-negative bacteria and teichoic acids in Gram-positive bacteria). This interaction destabilizes membrane integrity, facilitates nanoparticle penetration, and enhances local ROS generation. Gram-negative bacteria such as *K. pneumoniae* are more sensitive due to thinner peptidoglycan layers, whereas Gram-positive bacteria exhibit slightly higher resistance because of thicker cell walls. In fungi like *C. albicans*, Ag⁺ ions and ROS disrupt ergosterol-containing membranes and interfere with mitochondrial function.

**Fig. 16:**
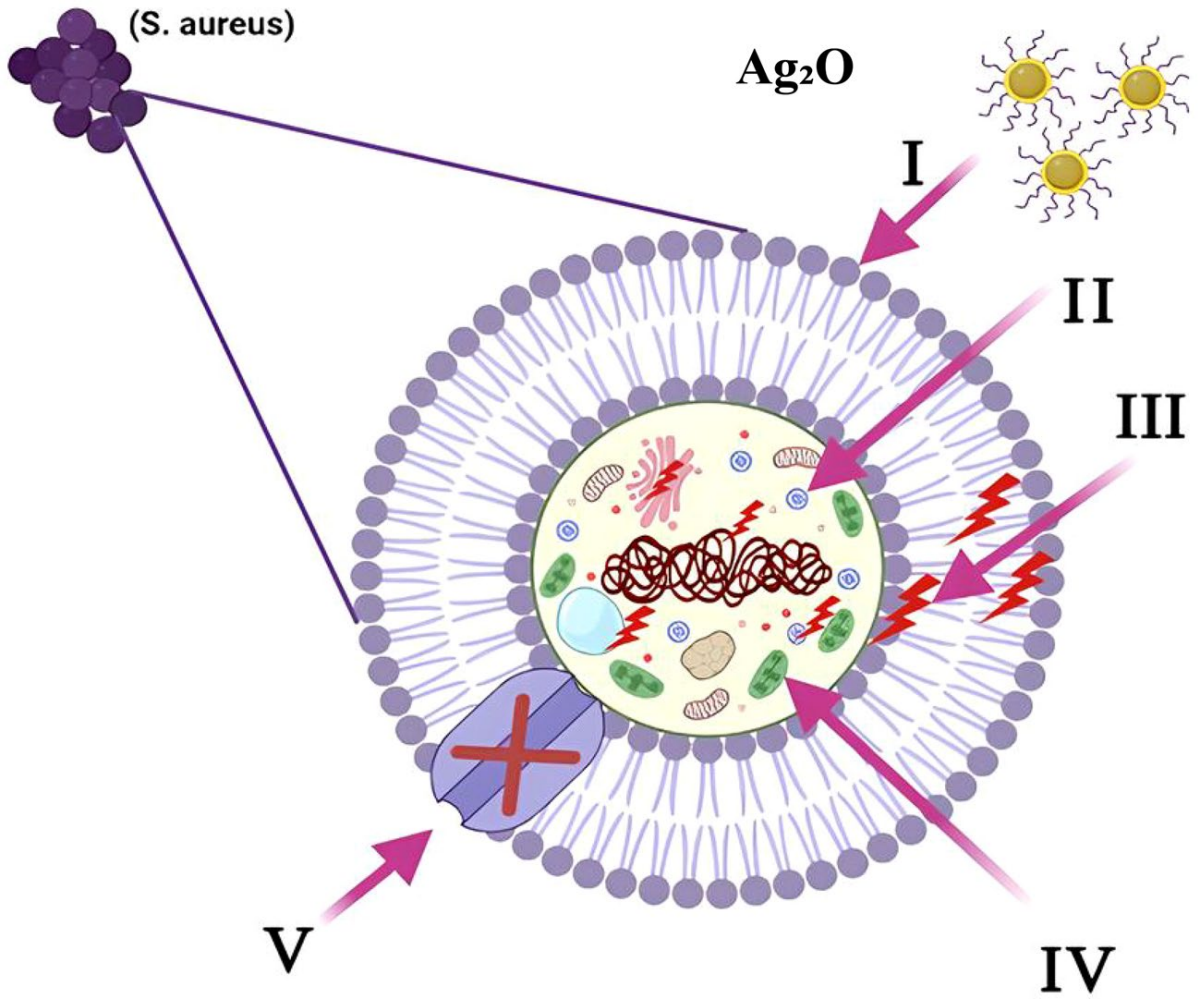
Schematic illustration of the primary antibacterial mechanisms of Ag_2_O: (I) Ag_2_O NPs adhere to the microbial cell surface, causing membrane disruption and altering transport functions. (II) Ag_2_O NPs penetrate the microbial cells, interacting with intracellular components and disturbing vital cellular activities. (III) Ag_2_O NPs induce the formation of reactive oxygen species (ROS), leading to oxidative stress and cellular damage. (IV) Ag_2_O NPs interfere with microbial signaling pathways, triggering programmed cell death. (V) Ag_2_O NPs disrupt ion transport across the microbial membrane, further impairing essential cellular functions.

Overall, the synergistic action of Ag^+^ leaching and ROS production explains the broad-spectrum antimicrobial performance of Ag_2_O/Z composites observed in this study, where the *Zea mays L*. matrix further enhances nanoparticle dispersion and stability, improving their bioactivity (Fig. 16).

## Conclusions

In this work *Zea mays L.* biomass was converted to value-added material by chemical deposition of a thin layer of Ag_2_O NPs on its surface with two ratios (e.g., 5 & 10%) to form novel cost-effective core-shell nanostructures. TEM and SEM images showed the precipitation of plate-like Ag_2_O NPs on the surface of spongy *Zea mays L.* biomass. Additionally, FTIR peaks of the synthesized core/shell nanostructures showed that the characteristic peaks of both Ag_2_O NPs and *Zea mays L.* appeared. Remarkably, at a pH of 9.0, 0.01 g of 10% Ag_2_O/Z nanocomposite removed 95.0% of MB. Ag₂O NPs exhibited the highest antibacterial activity against *K. pneumoniae* (24.7 ± 0.6 mm), followed by moderate inhibition of S. *aureus* (16.7 ± 0.6 mm) and *B. subtilis* (14.7 ± 0.6 mm). These results confirm that Ag₂O is the primary antimicrobial agent, but incorporation with the *Zea mays L*. matrix modifies and, in some cases, enhances its effectiveness against Gram-positive bacteria. Silver-coated *Zea mays L.* nanocomposites offer a biocompatible and biodegradable solution for antimicrobial applications and wastewater treatment. Their porous structure aids in contaminant adsorption, while silver’s antimicrobial properties effectively reduce pathogens. This cost-effective approach utilizes agricultural waste, enhancing filtration and scalability for larger treatment facilities. Overall, these nanostructures present a sustainable method for addressing health and environmental challenges. While silver-based nanomaterials offer benefits, they pose a risk of Ag⁺ ion leaching, which could lead to eco-toxicity in soil or water. Although the *Zea mays L.* matrix may enhance nanoparticle stability and potentially reduce uncontrolled ion release, this risk still exists and cannot be ignored. Future work should therefore evaluate silver ion release and environmental impact to ensure the safe and sustainable application of Ag₂O/Z nanocomposites.

## Supplementary Information

Below is the link to the electronic supplementary material.

Supplementary Material 1

## Data Availability

The datasets used and analyzed during the current study are available from the corresponding author on reasonable request.
