# Supplementary material for: Silver-coated Zea mays L. nanocatalyst for efficient Azo dye photodegradation and antimicrobial applications
Source: Sci Rep. 2025 Oct 30;15:37931. doi: 10.1038/s41598-025-22961-9 (PMC12575801; doi:10.1038/s41598-025-22961-9)
Supplement: Supplementary file 1 — Supplementary Material 1 [file 41598_2025_22961_MOESM1_ESM.docx]

**Silver-Coated** ***Zea mays L.*** **Nanocatalyst for Efficient Azo Dye Photodegradation and Antimicrobial Applications**

***Walaa M. Abd El‐Gawad ^1^, Karim Elmaghraby ^2,3*^, Ahmed M. El-Khawaga ^4*^***

*^1^Polymers and Pigments Dept., National Research Centre, Dokki,* 12622*, Cairo*, Egypt.

*^2^Department of Botany, Faculty of Science, Tanta University, 31527- Tanta, Egypt.*

*^3^Department of Biotechnology, Chemistry and Pharmacy, University of Siena, 53100 - Siena, Italy.*

*^4^Department of Basic Medical Sciences, Faculty of Medicine, Galala University, Galala City 43511, Suez, Egypt.*

***Corresponding Authors’ E-mails:**

[**ahmed.elkhawaga@gu.edu.eg**](mailto:ahmed.elkhawaga@gu.edu.eg) **and** [**k.elmaghraby@student.unisi.it**](mailto:k.elmaghraby@student.unisi.it)


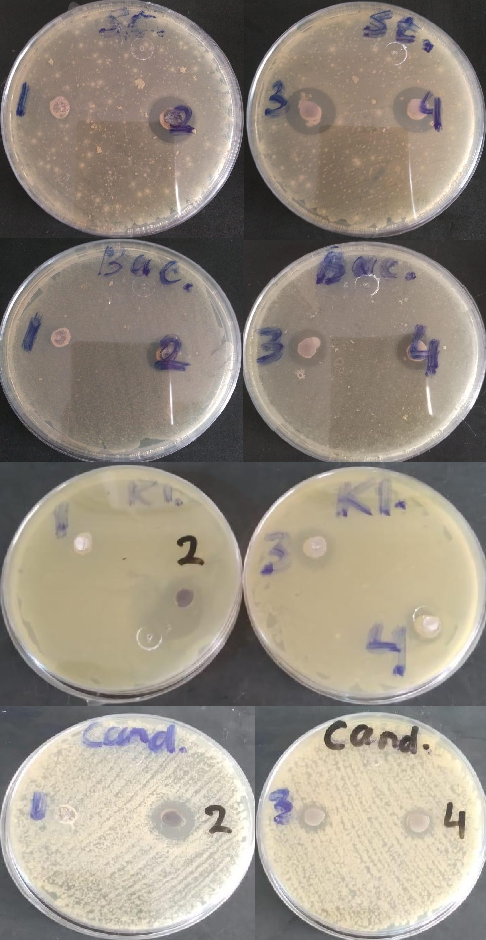


**Figure S1. Antibacterial and antifungal activities of *Zea mays L.;* Ag_2_O; 5% Ag_2_O/Z and 10% Ag_2_O nanocomposite on: (a) *Staphylococcus aureus*, (b) *Bacilus Subtits,* (c) *Klebsiella pneumoniae* and (d) *Candida albicans* measured as ZOI (mm). 1 =  *Zea mays L.*; 2 =  Ag_2_O; 3 = 5% Ag_2_O/Z, 4 = 10% Ag_2_O/Z.**
